# The CP-STPG3-AS1 axis regulates lipoylation and cuproptosis in castration-resistant prostate cancer

**DOI:** 10.3389/fonc.2026.1989277

**Published:** 2026-10-22

**Authors:** Jia Wei He, Pei Zhen Li, Zhen Zhao Zhang

**Affiliations:** 1 Department of Urology, The Affiliated Dongguan Songshan Lake Central Hospital, Guangdong Medical University, Dongguan, Guangdong, China; 2 Department of Orthopedics, Dongguan Eighth People’s Hospital, Dongguan, Guangdong, China; 3 Department of Rehabilitation Medicine, The Affiliated Dongguan Songshan Lake Central Hospital, Guangdong Medical University, Dongguan, Guangdong, China

**Keywords:** ceruloplasmin, cuproptosis, lipoylation, prostate cancer, STPG3-AS1

## Abstract

**Background:**

Prostate cancer (PCa), particularly metastatic castration-resistant PCa (mCRPC), remains therapeutically challenging. Cuproptosis, a copper-dependent cell death driven by lipoylated tricarboxylic acid cycle proteins, is implicated in PCa progression, but whether ceruloplasmin (CP), a key copper-binding glycoprotein, modulates cuproptosis in PCa and the underlying mediators remain unknown.

**Methods:**

We integrated bioinformatic analysis of TCGA-PRAD data (550 PCa, 52 normal tissues) with *in vitro* validation in two androgen-independent CRPC cell lines: PC-3 cells received exogenous CP gradients, while 22Rv1 cells underwent CP knockdown and rescue experiments with qPCR, Western blot, MitoSOX flow cytometry, and CCK-8 assays.

**Results:**

Eighty CP-cuproptosis intersection genes were identified. Cuproptosis core genes (FDX1, DLST, LIAS, DLD) were significantly upregulated in PCa (all p<0.001), while CP showed no differential expression but weak positive correlations with these genes (r=0.205-0.300). Exogenous CP concentration-dependently reduced PC-3 metabolic activity and induced apoptosis-related morphology. In 22Rv1 cells, CP knockdown downregulated lncRNA STPG3-AS1, upregulated cuproptosis-related markers (FDX1, DLAT, lipoylated proteins) and mitochondrial ROS, and inhibited proliferation; STPG3-AS1 overexpression reversed all these effects. No gene showed independent prognostic value.

**Conclusion:**

STPG3-AS1 mediates CP-regulated lipoylation and cuproptosis-associated molecular changes in CRPC cells, identifying a novel CP-STPG3-AS1-cuproptosis regulatory axis with therapeutic potential for advanced prostate cancer.

## 1 Introduction

Prostate cancer (PCa) is the most common urological malignancy and a leading cause of male cancer-related mortality worldwide, imposing a heavy global clinical burden (1, 2). Globally, PCa was the second most frequently diagnosed cancer among men in 2022, with approximately 1.47 million new cases and 397,000 deaths (3). In the United States, it remains the most commonly diagnosed cancer among men and the second leading cause of male cancer death, with an estimated 333,830 new cases (15.8% of all new cancer cases) and 36,320 deaths projected for 2026 (4). Clinical management of advanced disease remains challenging: docetaxel, the first-line taxane, achieves a ≥50% PSA decline in only approximately 45% of patients with metastatic castration-resistant PCa (mCRPC) in the landmark TAX 327 trial (5), and although the therapeutic landscape has since expanded to include androgen receptor pathway inhibitors (e.g., enzalutamide, abiraterone), the second-line taxane cabazitaxel, the radioligand agent [177Lu]Lu-PSMA-617, PARP inhibitors and immunotherapy—options endorsed by current guidelines (6, 7)—acquired resistance and eventual progression remain inevitable (8, 9). Despite these advances, the lack of mechanistic insights into key biological pathways underlying PCa progression limits the development of effective targeted therapies, driving the need to explore novel regulatory mechanisms and potential therapeutic targets for this disease (10–13).

Copper homeostasis, maintained by transporters (CTR1, ATP7A/B), chaperones (GSH, CCS, ATOX1) and the copper-binding glycoprotein ceruloplasmin (CP), is frequently disrupted in PCa, with elevated serum and tumor copper levels linked to advanced disease stage, high pathological grade and poor clinical outcomes (14–16). This homeostatic imbalance is closely associated with cuproptosis, a unique copper-dependent regulated cell death pathway characterized by copper binding to lipoylated tricarboxylic acid (TCA) cycle components (e.g., DLAT, DLST), which triggers protein oligomerization, Fe–S protein loss and subsequent proteotoxic stress-induced cell death (17, 18). Classic core regulators of cuproptosis, including FDX1 (a mediator of Cu²^+^ reduction) and lipoylated TCA cycle enzymes (DLST, LIAS, DLD), have been implicated in PCa pathogenesis, and emerging studies have identified cuproptosis-related prognostic signatures and tentative therapeutic strategies for PCa. However, the biological crosstalk between CP and the cuproptosis pathway in PCa remains poorly elucidated; while preliminary correlations between CP and cuproptosis core genes have been suggested, no direct experimental evidence confirms a regulatory relationship, and the molecular mediators (e.g., long non-coding RNAs, lncRNAs) underlying this interaction have not been identified to date (19). Notably, the cuproptosis field has advanced rapidly in recent years. Beyond the seminal discovery that copper induces cell death by binding to lipoylated tricarboxylic acid (TCA) cycle enzymes, cuproptosis has been shown to enhance docetaxel chemosensitivity through the DLAT/mTOR axis in PCa, enzalutamide sensitizes CRPC cells to copper-mediated cell death, and AR-targeted therapies have recently been demonstrated to transcriptionally activate FDX1, thereby priming prostate cancer cells for cuproptosis (20). Cuproptosis-related lncRNA signatures further stratify PCa prognosis, biochemical recurrence and immune microenvironment, and comprehensive reviews have consolidated the mechanistic landscape and therapeutic potential of cuproptosis across malignancies (21). Together, these advances underscore the clinical relevance of copper-cuproptosis crosstalk in PCa, and highlight the unmet need to identify upstream copper-homeostasis regulators, such as CP, and the lncRNA mediators of their effects.

Prior research into CP and cuproptosis in PCa is limited by several critical gaps: most studies rely solely on bioinformatic correlation analyses without *in vitro* functional validation, use a single PCa cell line with no assessment of cuproptosis-specific molecular markers [e.g., lipoylated proteins, mitochondrial reactive oxygen species (mtROS)], and lack knockdown or rescue experiments to confirm causal relationships (22–24). Although CP is a key regulator of copper homeostasis, its expression pattern in PCa tissues and its functional effects on cuproptosis-related cell behavior remain unclear, and no studies have explored whether lncRNAs mediate the regulatory effect of CP on the cuproptosis pathway in CRPC cells (25–27). Additionally, existing cuproptosis-related research in PCa has not fully addressed the role of copper homeostasis regulators in modulating cuproptosis activity, leaving a critical knowledge gap in understanding how systemic copper metabolism interfaces with cell-intrinsic cuproptosis in PCa progression.

To address these gaps, the present study first integrated bioinformatic analyses of the TCGA-PRAD dataset (550 PCa and 52 normal tissues) to identify intersection genes between CP-associated genes and the cuproptosis-related gene set, analyze their expression patterns and correlation, and evaluate their prognostic value in PCa (28–30). We then performed *in vitro* experiments using two androgen-independent CRPC cell lines (PC-3 and 22Rv1) to validate the functional effects of CP on PCa cells: PC-3 cells were used for a concentration gradient experiment to assess the impact of exogenous CP on cell metabolic activity and apoptosis-related morphology, while 22Rv1 cells were used for CP knockdown and rescue experiments to verify the regulatory effect of CP on cuproptosis core molecules (FDX1, DLAT, lipoylated proteins) and the mediating role of specific lncRNAs (STPG3-AS1, LIPE-AS1). We also detected mtROS levels to link CP regulation to cuproptosis-related oxidative stress. The primary objective of this study was to verify that STPG3-AS1 mediates ceruloplasmin-regulated lipoylation and cuproptosis in castration-resistant prostate cancer, and to provide experimental evidence for this regulatory mechanism (31). The findings of this study aim to fill critical gaps in the current understanding of copper homeostasis and cuproptosis in PCa, and lay a foundational framework for future mechanistic research into CP-mediated cuproptosis regulation as a potential therapeutic strategy for CRPC.

## 2 Method

### 2.1 Data sources

The cuproptosis-related gene set (1,046 genes, including the classic core regulators FDX1, DLST, LIAS, DLD and DLAT) was assembled by pooling all genes annotated as cuproptosis-related in three independent sources: (i) two cuproptosis-related gene sets of the Gene Set Enrichment Analysis (GSEA) platform (array platform accessions GPL11670 and GPL14663; https://www.gsea-msigdb.org/gsea/msigdb), retrieved on the dates specified in **Supplementary Table S1**; (ii) GeneCards (https://www.genecards.org; retrieved on the date specified in **Supplementary Table S1**), using a keyword query for “cuproptosis”; and (iii) published cuproptosis-related gene collections, including the core cuproptosis regulators reported by Tsvetkov et al. (2022) and complementary cuproptosis-related gene sets. All genes returned by the source queries were pooled without additional expression-based filtering to avoid selection bias, and duplicate genes across sources were removed according to official gene symbols. The complete 1,046-gene list, with the sources of each gene annotated, is provided as **Supplementary Table S1**. CP-associated genes for protein-protein interaction (PPI) network construction were obtained primarily from a GeneCards search for “ceruloplasmin” (https://www.genecards.org; retrieved on the date specified in **Supplementary Table S1**; no relevance-score threshold was applied, and all genes returned by the query were retained), supplemented by protein interaction data from the STRING database (https://string-db.org; v12.0, confidence score > 0.4); duplicate genes were removed by gene symbol. The TCGA-PRAD dataset, including gene expression profiles of 550 prostate cancer (PCa) and 52 normal tissues as well as clinical prognosis data of 492 patients, was retrieved from the UCSC Xena or GEPIA2 platform, and all gene expression data were subjected to batch correction via the ComBat method before subsequent bioinformatic analyses.

### 2.2 Bioinformatics analysis

Venny 2.1 was used to identify intersection genes between CP-associated genes and the cuproptosis-related gene set, with an expression heatmap generated by the R package pheatmap. For target genes (CP, FDX1, DLST, LIAS, DLD), expression differences between tumor and normal tissues were analyzed by the Wilcoxon rank-sum test after quality control and log_2_(count+1) transformation, with violin plots visualized; Differentially expressed genes (DEGs) between tumor and normal tissues were independently screened with thresholds of FDR<0.05 and |logFC|>2; these DEGs were not involved in the intersection analysis described below. Pearson correlation coefficient was used to analyze gene expression correlations, and patients were stratified into high/low expression groups by median expression for Kaplan-Meier survival analysis and Log-rank test. The R package clusterProfiler was applied for GO and KEGG enrichment analyses (P<0.05) of intersection genes; these genes were further imported into STRING (confidence>0.7) to construct a PPI network, and core node genes (Degree>12) were screened via the CytoHubba plugin (Degree algorithm) in Cytoscape.

### 2.3 Experimental materials and grouping

Two androgen-independent castration-resistant prostate cancer (CRPC) cell lines (PC-3 and 22Rv1) were purchased from ATCC. The cell lines were authenticated by short tandem repeat (STR) genotyping by ATCC, and the authentication report was provided with the cell lines. The cells were cultured in RPMI-1640 medium containing 10% fetal bovine serum and 1% penicillin-streptomycin at 37 °C with 5% CO_2_ and saturated humidity. Key reagents included human serum ceruloplasmin (CP; purchased from Sigma-Aldrich (St. Louis, MO, USA; catalog no. C4519), purity≥95% as reported by the manufacturer and confirmed by SDS-PAGE; endotoxin level<0.1 EU/μg (as certified by the manufacturer; see certificate of analysis); the native copper-loaded (holo-CP) form, with a copper content of 1.0-2.5 μg/mg protein as specified by the manufacturer), CCK-8 kit, Hoechst 33258 staining solution, MitoSOX Red probe, specific siRNAs (the most effective si-CP sequence: 5’-GCUUGUAUUCCAUGGGCUUTT-3’), oe-STPG3-AS1 and oe-LIPE-AS1 overexpression plasmids; the overexpression plasmids were verified by double restriction enzyme digestion and Sanger sequencing, and primary antibodies against cuproptosis-related molecules; major instruments included a CO2 incubator, flow cytometer, fluorescence microscope, and Western blot electrophoresis system. PC-3 cells were treated with 0, 50 and 100 μg/mL CP for 24 h and 48 h (Hoechst 33258 staining was performed at 48 h), with concentrations selected based on physiological serum CP levels, previous studies and pilot experiments; the CP stock solution was freshly diluted in complete culture medium before each treatment. 22Rv1 cells were assigned to 6 groups (3 replicates each): blank control, si-NC, si-SLC31A1, si-CP, si-SLC31A1+oe-LIPE-AS1, and si-CP+oe-STPG3-AS1, with transfection performed via Lipofectamine 3000 and subsequent experiments conducted 48 h post-transfection.

The two cell lines represent complementary molecular subtypes of CRPC. PC-3, established from a bone metastasis of a prostatic adenocarcinoma, is androgen-independent and AR-negative, with PTEN loss and TP53 mutation, and represents the AR-independent, AR-null subtype. 22Rv1, derived from the castration-recurrent CWR22 xenograft, expresses full-length androgen receptor together with the constitutively active AR splice variant AR-V7 and grows without androgen supplementation, representing the AR-dependent, AR-V7-driven subtype. Both lines are therefore androgen-independent and are widely accepted *in vitro* models of CRPC, and their combined use captures the AR-phenotypic heterogeneity of clinical CRPC.

STPG3-AS1, LIPE-AS1 and AC005790.1 were selected from published cuproptosis-related lncRNA signatures in prostate cancer: STPG3-AS1 and LIPE-AS1 are both members of a validated six-lncRNA cuproptosis-related prognostic signature, and LIPE-AS1/AC005790.1 were additionally highlighted in cuproptosis-related lncRNA analyses of the PCa immune microenvironment and biochemical recurrence. There is no known direct biological relationship between LIPE-AS1 and STPG3-AS1; they are distinct lncRNA species included here to serve as parallel, specificity readouts. Specifically, STPG3-AS1 was prioritized as the candidate CP-regulated lncRNA because of its validated membership in the published cuproptosis-related lncRNA signature for PCa and its reported associations with PCa prognosis, biochemical recurrence and the immune microenvironment; this prioritization was confirmed by the CP-knockdown experiments described below, in which CP silencing selectively downregulated STPG3-AS1, whereas LIPE-AS1 (together with AC005790.1) was assigned to the SLC31A1 (CTR1, the principal copper-import transporter) arm as a specificity control. This paired design tests whether distinct copper-homeostasis genes regulate distinct cuproptosis-related lncRNAs, and whether the CP-STPG3-AS1 relationship is lncRNA-specific rather than a general effect of perturbing copper transport.

Mycoplasma contamination was strictly monitored during the entire cell culture process to ensure the reliability of experimental results. The PC-3 and 22Rv1 cells were tested for mycoplasma every 2 weeks using a commercial mycoplasma PCR detection kit (Takara, Japan) in accordance with the manufacturer’s instructions. All cell lines used in the experiments were confirmed to be mycoplasma-negative by PCR detection, and no mycoplasma infection was found in any experimental stage. In addition, all key experiments (including CP concentration gradient assays in PC-3 cells, CP/SLC31A1 knockdown and rescue experiments in 22Rv1 cells, mtROS detection, and cell proliferation assays) were independently repeated three times using mycoplasma-free cell cultures, and the experimental findings were consistently reproduced with no significant differences in the results of repeated experiments (all P>0.05 for the consistency test of experimental repeatability).

### 2.4 Experimental detection methods

For PC-3 cells, CCK-8 assay was used to detect relative metabolic activity (reflecting proliferation-associated mitochondrial dehydrogenase activity) at 24 h and 48 h, and Hoechst 33258 staining was performed to observe apoptosis-related morphological changes at 48 h and calculate the percentage of cells with nuclear condensation/fragmentation. For 22Rv1 cells at 48 h post-transfection, qPCR (2^-^ΔΔCt method, GAPDH as internal reference) was used to detect the expression of three lncRNAs (LIPE-AS1, AC005790.1, STPG3-AS1); Western blot (BCA protein quantification, β-actin as internal reference) was applied to measure the expression of CP, FDX1, DLAT and lipoylated proteins (a specific cuproptosis marker); MitoSOX Red flow cytometry was used to detect the positive rate of mitochondrial reactive oxygen species (mtROS); and CCK-8 assay was performed to calculate cell proliferation inhibition rate at 24 h, 48 h and 72 h.

### 2.5 Statistical analysis

All experimental data were expressed as mean ± standard deviation (Mean ± SD) from three independent biological replicates (each with three technical replicates) for all *in vitro* assays, including CCK-8, Hoechst 33258 staining, qPCR, Western blot, and MitoSOX Red flow cytometry. All experiments were performed in triplicate for each group as described in the experimental grouping, and analyzed using SPSS 26.0 and GraphPad Prism 9.0 software. Independent-samples t-test was used for comparisons between two groups, and one-way analysis of variance (ANOVA) followed by LSD *post-hoc* test for multiple group comparisons. Pearson correlation analysis was applied to evaluate gene expression correlations, and a P value<0.05 was considered statistically significant.

## 3 Results

### 3.1 Intersection identification and expression profiles of CP-associated genes and cuproptosis-related genes

Differentially expressed genes (DEGs) were screened from the TCGA-PRAD dataset with strict thresholds (FDR<0.05, |logFC|>2), yielding a total of 320 DEGs including 240 downregulated and 80 upregulated genes in prostate cancer (PCa) tissues. Intersection analysis between CP-associated genes and the cuproptosis-related gene set (1,046 genes, containing classic core regulators) via Venny 2.1 identified 80 overlapping genes, which were implicated in CP-cuproptosis crosstalk. Differential expression analysis in TCGA-PRAD revealed that, among the intersection genes, 58 were downregulated (logFC: -9.92 to -1.02) and 22 were upregulated (logFC: 1.02 to 3.82) in PCa tissues, consistent with the expression trend of key intersection genes. Functional annotation revealed that these genes were involved in copper ion metabolism, cell death regulation and signal transduction, with cuproptosis-related genes (e.g., GSTM3, SLC7A11) included, supporting their functional relevance in linking CP and cuproptosis. Hierarchical clustering heatmap further displayed clear separation of expression profiles between downregulated (e.g., SCO2, GOLM1) and upregulated (e.g., CHI3L1, PAX8) intersection genes in PCa and normal tissues (**Table 1**)(**Figure 1**).

**Table 1:** TCGA-PRAD expression statistics of representative CP-cuproptosis crosstalk genes.

Gene symbol	logFC	logCPM	P Value	FDR
MPO	-2.87	5.62	3.21×10^-^¹^9^	1.83×10^-^¹^7^
HP	-3.15	6.18	7.59×10^-^²³	5.92×10^-^²¹
SERPINA1	-1.93	7.04	2.07×10^-^¹²	8.66×10^-^¹¹
HMOX1	2.08	4.89	5.13×10^-^¹^5^	3.02×10^-^¹³
LTF	-2.54	5.37	9.44×10^-^¹^8^	4.98×10^-^¹^6^
ALB	-3.52	8.26	1.12×10^-^²^8^	1.05×10^-^²6
FXN	-1.26	6.53	4.78×10^-8^	1.56×10^-7^
SLC40A1	-1.79	5.91	6.35×10^-^¹^4^	3.11×10^-^¹²
ATP7B	-2.01	4.75	8.92×10^-^¹³	3.87×10^-^¹¹
ATP7A	-1.54	5.22	2.69×10^-^¹¹	9.83×10^-^¹^0^
SCO2	-2.33	5.86	5.47×10^-^¹7	2.59×10^-^¹5
SERPINA3	2.41	6.03	3.82×10^-^²0	2.19×10^-^¹8
CHI3L1	2.76	5.59	1.64×10^-^²2	1.18×10^-^²0
GOLM1	-1.88	6.47	7.93×10^-^¹5	3.78×10^-^¹3
F5	-2.15	4.92	9.06×10^-^¹4	4.12×10^-^¹2
CXCL10	2.29	5.16	4.35×10^-^¹8	2.31×10^-^¹6
AOC1	-1.42	5.33	6.81×10^-^¹0	2.09×10^-9^
S100A2	1.97	5.78	8.25×10^-^¹6	4.27×10^-^¹4
CA12	-2.61	5.04	2.93×10^-^¹9	1.67×10^-^¹7
PDGFC	1.58	4.83	3.57×10^-^¹1	1.24×10^-9^
AMBP	-2.45	6.29	5.18×10^-^²1	3.05×10^-^¹9
GC	-3.02	7.65	8.74×10^-^²6	7.91×10^-^²4
FGB	-2.78	6.83	1.36×10^-^²4	1.12×10^-^²2
HBB	-1.69	4.67	7.22×10^-^¹2	2.85×10^-^¹0
SLC31A2	-1.99	5.41	6.03×10^-^¹5	2.89×10^-^¹3
PAX8	2.84	5.96	2.01×10^-^²5	1.89×10^-^²3

The listed genes include members of the 80-gene CP-cuproptosis intersection set as well as CP-associated genes that were differentially expressed in PCa tissues; the complete cuproptosis-related and CP-associated gene sets are provided in **Supplementary Tables S1, S2**, respectively.

**Figure 1:**
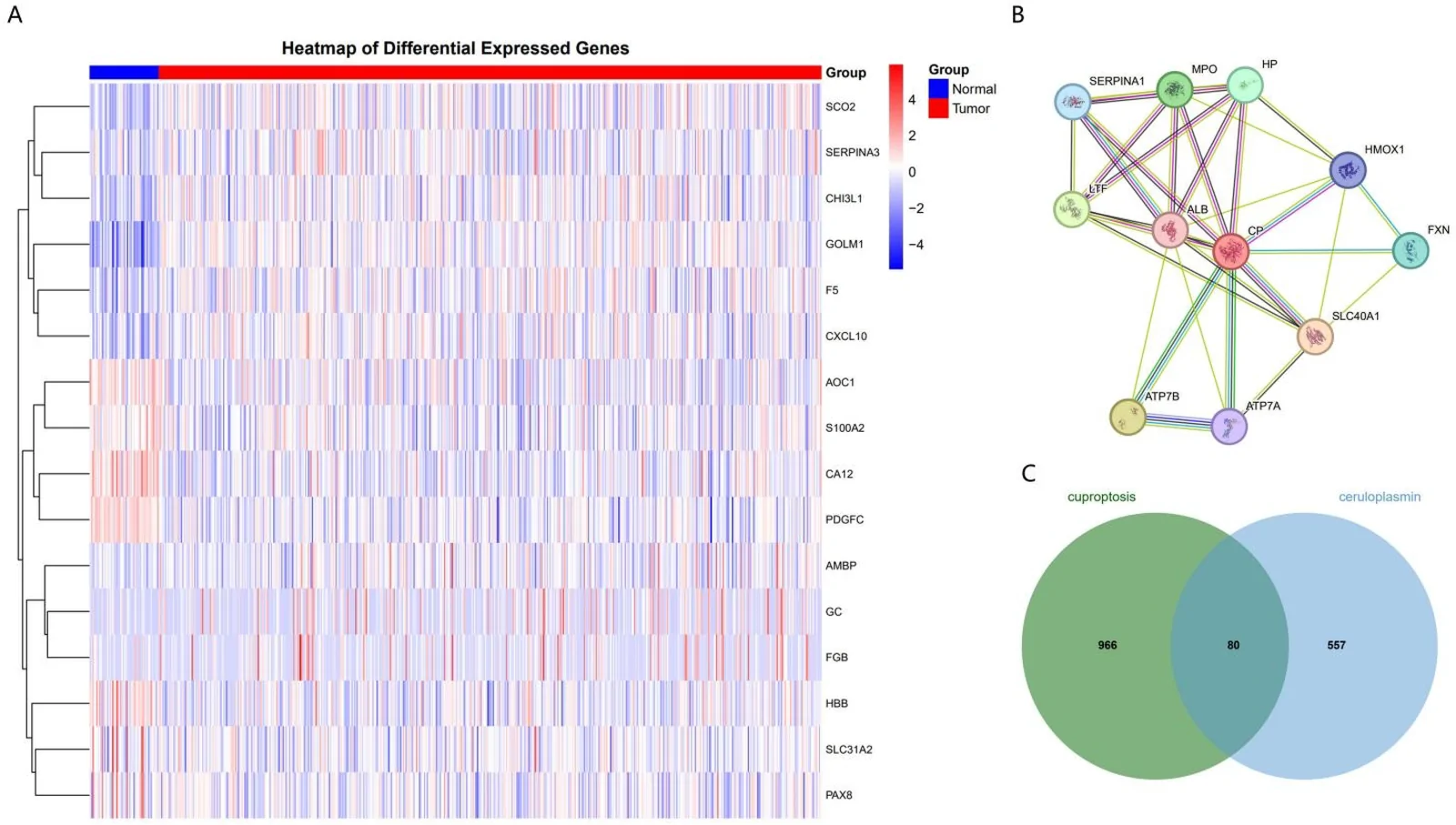
Identification of intersection genes between CP-associated genes and core cuproptosis genes, and their expression profiles: **(A)** Heatmap showing the hierarchical clustering expression patterns of intersection genes between CP-associated genes and core cuproptosis genes in prostate cancer (tumor) and normal tissues. **(B)** Protein-protein interaction (PPI) network of CP-related genes. **(C)** Venn diagram illustrating the overlapping region between CP-associated genes and the core cuproptosis gene set, with a total of 80 intersection genes identified.

### 3.2 Expression differences of CP and classic cuproptosis core genes in PCa vs. normal tissues

After data quality control and log_2_(count+1) transformation, Wilcoxon rank-sum test was used to analyze the expression differences of CP and classic cuproptosis core regulators (FDX1, DLST, LIAS, DLD) between PCa and normal tissues. CP expression showed no statistically significant difference between the two groups (p=0.46), with overlapping expression distributions in violin plots, indicating CP was not a direct transcriptional indicator of PCa malignancy. In contrast, all four cuproptosis core genes were significantly upregulated in PCa tissues (all p<0.001): DLD (p=1.31e−05), DLST (p=3.34e−06), FDX1 (p=0.000458) and LIAS (p=0.000232). Violin plots demonstrated a distinct shift of these four genes toward higher log_2_-transformed expression values in PCa, suggesting their potential involvement in cuproptosis pathway dysregulation during PCa development (**Figure 2**).

**Figure 2:**
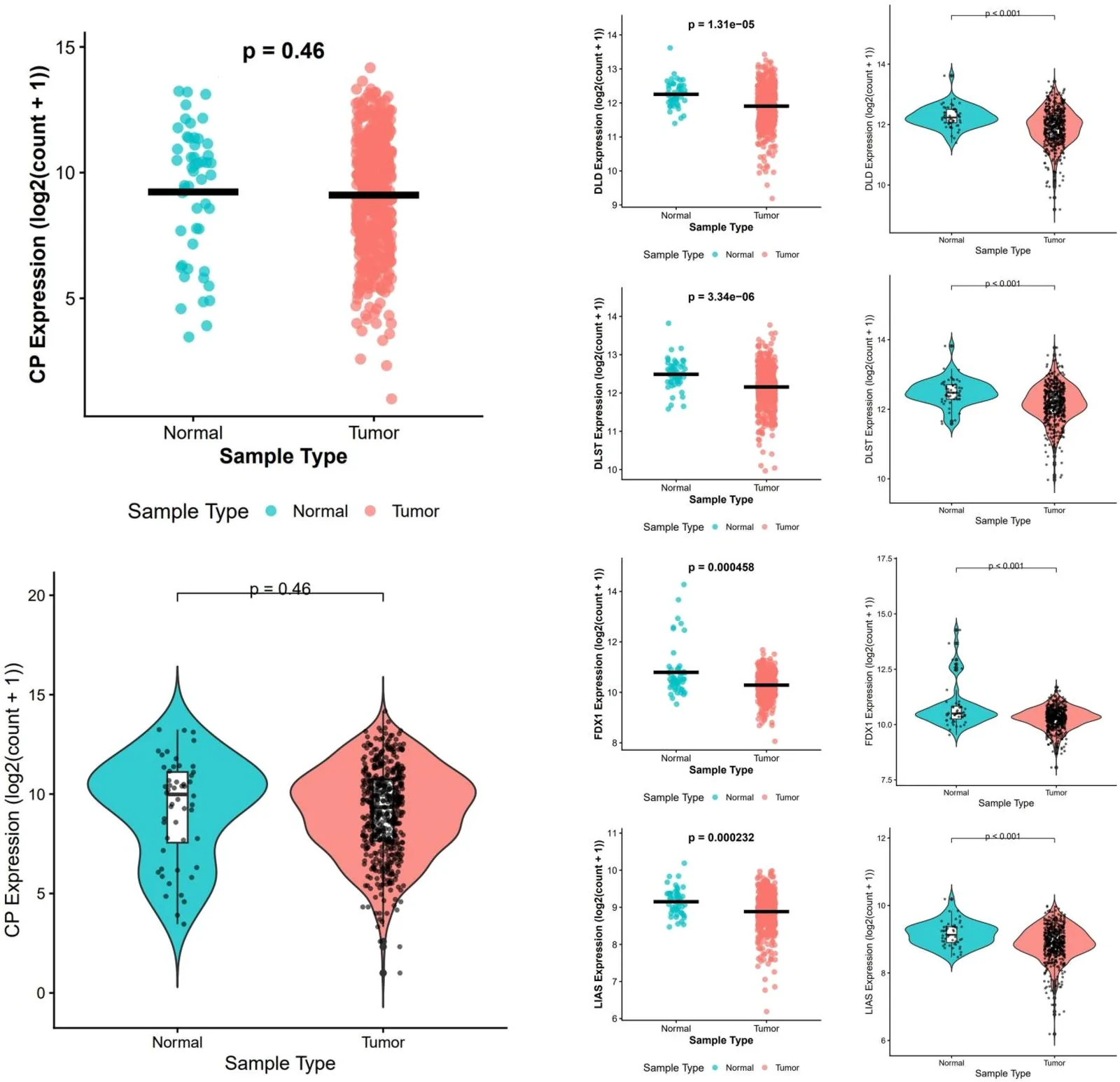
Expression differences of CP and key cuproptosis genes between prostate cancer tissues and normal tissues.

### 3.3 Correlation between CP and cuproptosis core genes and their prognostic value in PCa

Pearson correlation analysis revealed statistically significant but weak positive correlations between CP and the four cuproptosis core genes in PCa tissues (all p<0.001), with the strongest correlation observed between CP and DLST (r=0.300), followed by DLD (r=0.253), LIAS (r=0.235) and FDX1 (r=0.205). Correlation scatter plots confirmed the mild positive linear relationships between CP and these core genes, implying a potential co-expression pattern without direct regulatory implications. Kaplan-Meier survival analysis (stratified by median gene expression) showed no statistically significant differences in overall survival (OS) between high and low expression groups of CP, FDX1, DLST, LIAS or DLD (all p>0.05). Univariate and multivariate Cox proportional hazards regression analyses (without clinical covariate adjustment) further verified the limited prognostic value of these genes, with all hazard ratio confidence intervals crossing 1 and p-values > 0.05. No single gene was identified as an independent prognostic biomarker for PCa patients (**Figure 3**).

**Figure 3:**
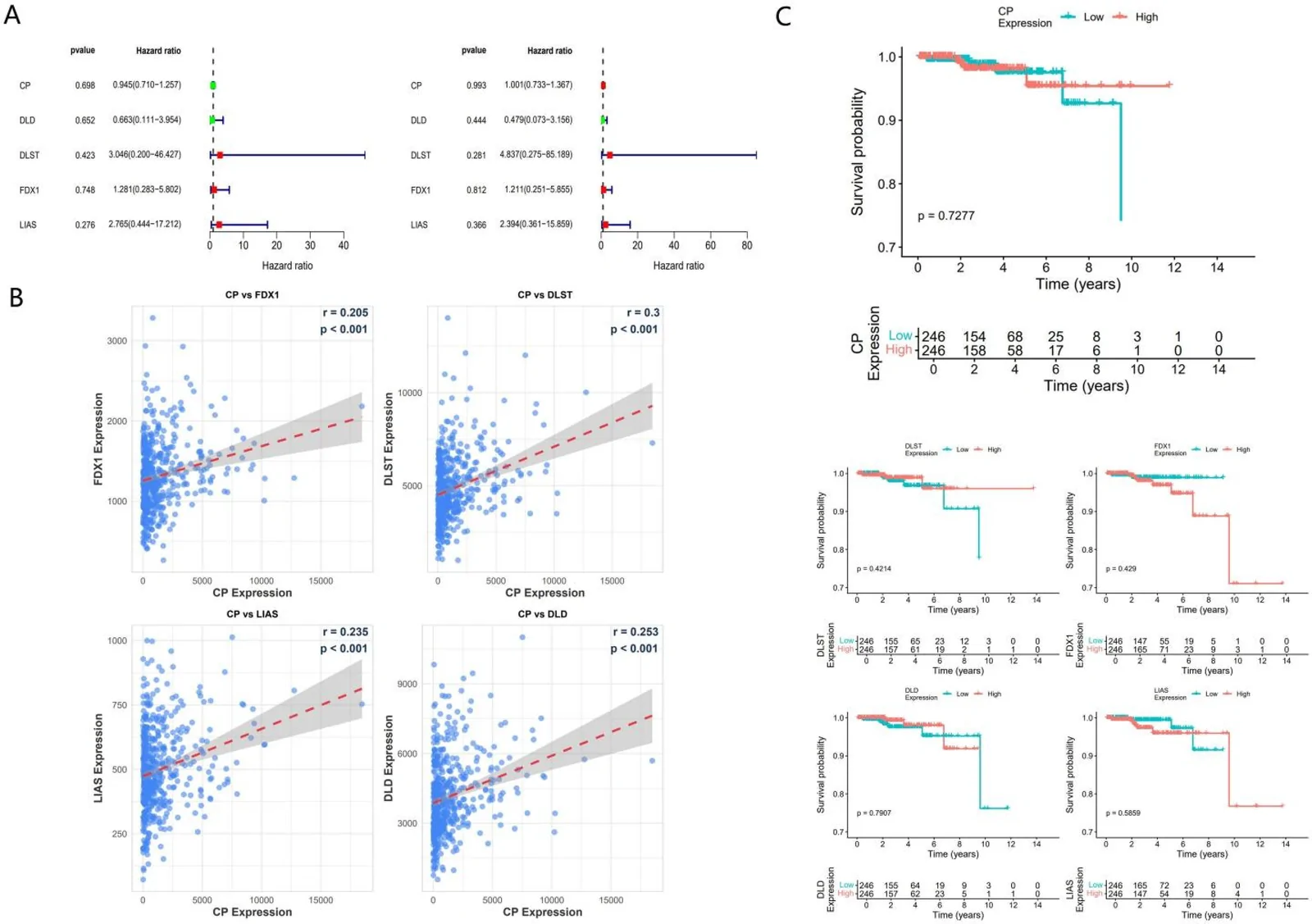
Correlation between CP and key cuproptosis genes, and their prognostic value in prostate cancer: **(A)** Forest plot from Cox proportional hazards regression analysis showing the impact of CP and key cuproptosis genes on overall survival (OS) of prostate cancer patients (no adjustment for clinical covariates). **(B)** Pearson correlation scatter plots demonstrating statistically significant but weak positive correlations between CP and FDX1 (r = 0.205), DLST (r = 0.300), LIAS (r = 0.235), and DLD (r = 0.253) (all p < 0.001). **(C)** Kaplan-Meier (KM) survival curves showing no statistically significant differences in OS between high and low expression groups of CP, FDX1, DLST, LIAS, or DLD (all p > 0.05). Patients were divided into high/low expression groups based on median expression, and the Log-rank test was used for survival analysis.

### 3.4 GO/KEGG enrichment and core nodes in PPI Network of CP-cuproptosis intersection genes

GO enrichment analysis (P<0.05) of the 80 intersection genes revealed distinct enrichment in three categories: biological process (BP) was predominantly enriched in intracellular copper ion homeostasis (GO:0006878) and copper ion homeostasis (GO:0055070); cellular component (CC) in blood microparticles (GO:0072562) and secretory granule lumen (GO:0034774); molecular function (MF) in copper ion binding (GO:0005507), heme binding (GO:0020037) and extracellular matrix structural constituent (GO:0005201). KEGG pathway enrichment analysis indicated potential associations of intersection genes with complement and coagulation cascades (hsa04610), nitrogen metabolism (hsa00910) and histidine metabolism (hsa00340), with moderate statistical significance after adjustment. PPI network construction (STRING, confidence>0.7) and core node screening via Cytoscape’s CytoHubba plugin (Degree algorithm) identified the top 10 core genes: APP (Degree=21), SOD1 (Degree=19), ATP7A (Degree=18), HIF1A (Degree=17), HMOX1 (Degree=17), JUN (Degree=15), SOD2 (Degree=15), MT1E (Degree=14), ATP7B (Degree=14) and ATOX1 (Degree=14). Copper ion transport and oxidative stress-related genes (ATP7A, ATP7B, HMOX1) were among these core nodes, further supporting the functional relevance of the PPI network in CP-cuproptosis crosstalk (**Figure 4**).

**Figure 4:**
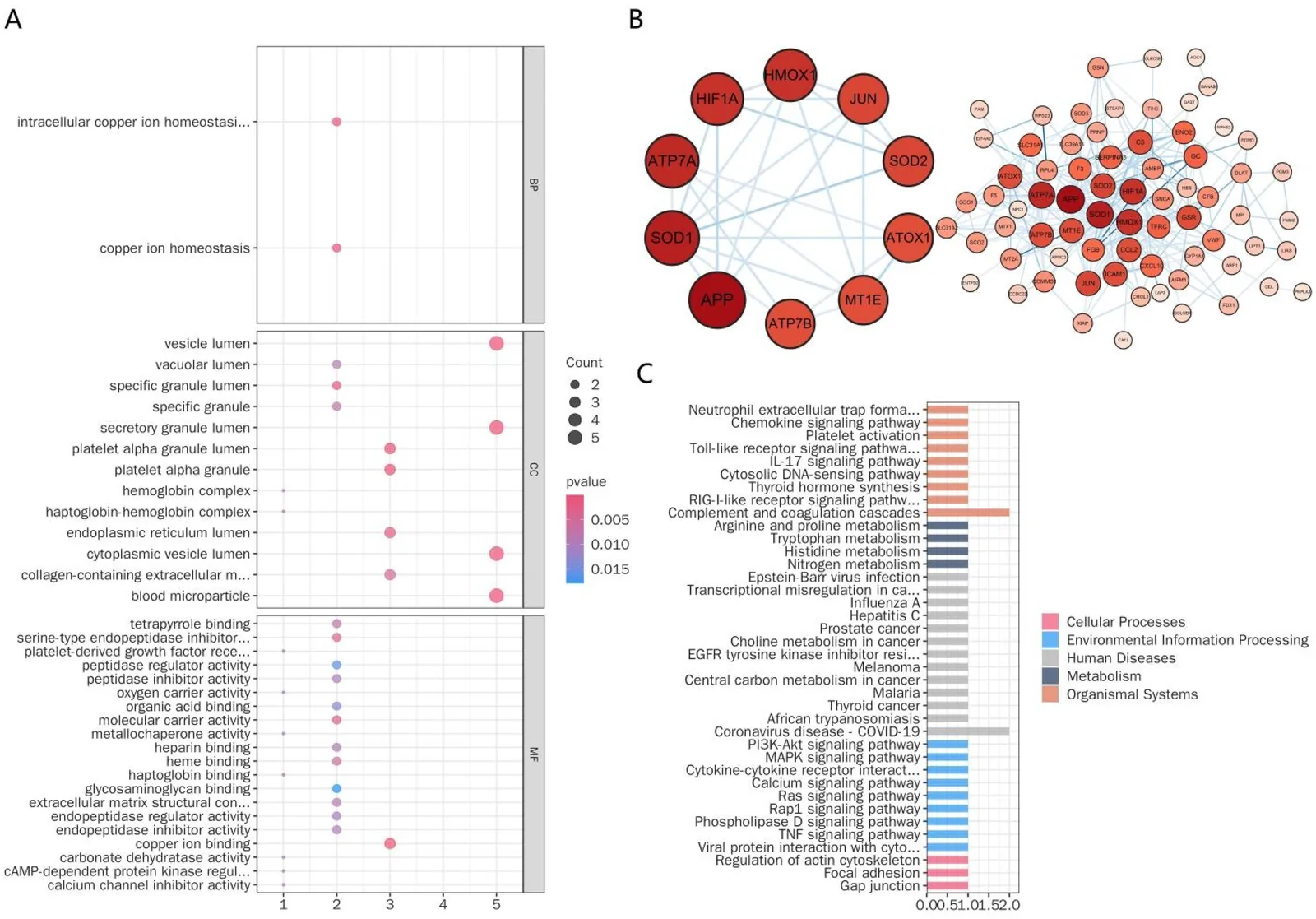
GO/KEGG enrichment analysis of CP-cuproptosis intersection genes and core nodes in their PPI network: **(A)** Bubble plot of Gene Ontology (GO) enrichment analysis (p < 0.05), showing the enrichment of intersection genes in biological processes (e.g., intracellular copper ion homeostasis, GO:0006878), cellular components (e.g., blood microparticles, GO:0072562), and molecular functions (e.g., copper ion binding, GO:0005507) — note: exploratory analysis, not experimentally validated. **(B)** Visualization of the PPI network of intersection genes (constructed via STRING with confidence > 0.7) and the top 10 core node genes (APP, SOD1, ATP7A, HIF1A, HMOX1, JUN, SOD2, MT1E, ATP7B, ATOX1) screened by the CytoHubba plugin (Degree algorithm). **(C)** Kyoto Encyclopedia of Genes and Genomes (KEGG) pathway enrichment analysis, indicating the association of intersection genes with pathways such as complement and coagulation cascades (hsa04610) and metabolic pathways (e.g., nitrogen metabolism, hsa00910) — note: exploratory analysis, not experimentally validated.

### 3.5 Effects of different CP concentrations on PC-3 cell metabolic activity and apoptosis-related morphology

CCK-8 assay results showed that exogenous CP treatment reduced the relative metabolic activity (reflecting proliferation-associated mitochondrial dehydrogenase activity) of PC-3 cells in a concentration- and time-dependent manner compared with the blank control group. At 24 h and 48 h, the relative metabolic activity of the 50 μg/mL CP group was 96.78% and 99.42% of the control, while that of the 100 μg/mL CP group was 88.08% and 91.26% of the control, respectively. Hoechst 33258 staining assay demonstrated that CP treatment significantly increased the percentage of PC-3 cells with apoptosis-related morphological changes (nuclear condensation/fragmentation) in a concentration-dependent manner: the blank control group had 9%-14% positive cells, the 50 μg/mL CP group 16%-20%, and the 100 μg/mL CP group 22%-31%. These results indicated that CP could mildly inhibit proliferation-associated metabolic activity and induce apoptosis-related morphological changes in PC-3 cells (**Figure 5**).

**Figure 5:**
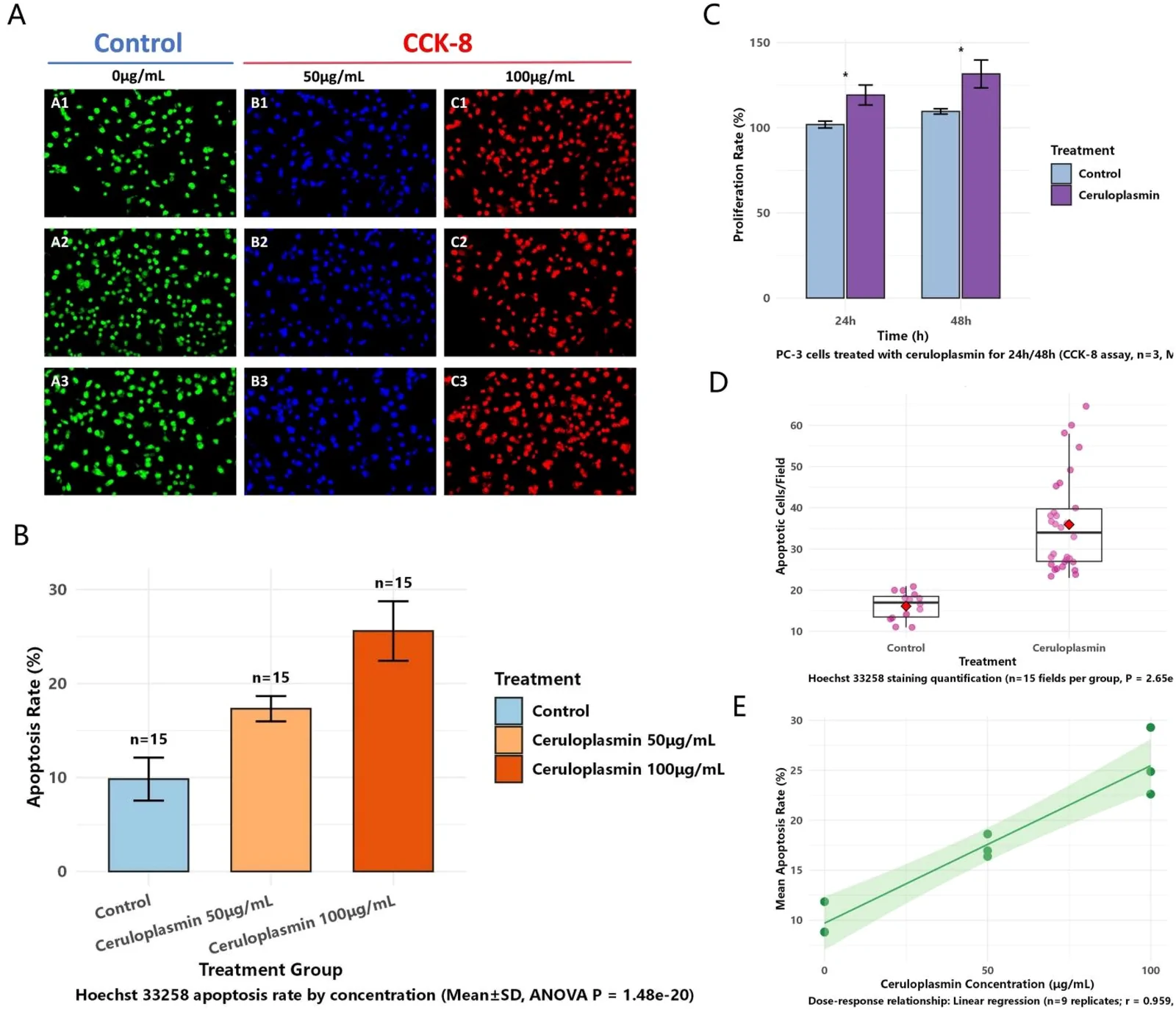
The effects of different concentrations of ceruloplasmin (CP) on the metabolic activity and apoptosis-related morphology of prostate cancer PC-3 cells, and the analysis of their correlation: **(A, D)** Hoechst 33258 staining (after 48 h of treatment) showing the morphology of PC-3 cells in the blank control group, low-concentration (50 μg/mL) CP group and high-concentration (100 μg/mL) CP group (A and D represent typical morphologies of different groups; arrows indicate cells with nuclear condensation/fragmentation, a morphological feature consistent with apoptosis). **(B)** Bar graph of the statistical analysis of the percentage of cells with apoptosis-related morphology in the blank control, low-concentration and high-concentration CP groups. **(C)** CCK-8 assay results showing the relative metabolic activity of PC-3 cells in each group at 24 h and 48 h (note: reflects mitochondrial dehydrogenase activity, not direct cell proliferation). **(E)** Scatter plot of the correlation between relative metabolic activity and percentage of cells with apoptosis-related morphology. Note: Hoechst 33258 staining is an observational method for apoptosis-related morphology, not a quantitative apoptosis assay; results reflect preliminary morphological changes rather than precise apoptotic cell counts.

### 3.6 CP knockdown induces cuproptosis-associated molecular changes and inhibits cell proliferation in 22Rv1 cells

qPCR detection of 22Rv1 cells at 48 h post-transfection showed that si-CP treatment significantly downregulated STPG3-AS1 expression by ≥30% (p<0.05), while si-SLC31A1 downregulated LIPE-AS1/AC005790.1 expression by ≥40% (p<0.05), confirming the specific regulatory relationship between CP/SLC31A1 and corresponding lncRNAs. Western blot analysis revealed that si-CP group exhibited a ≥50% upregulation of FDX1, DLAT and lipoylated proteins (a specific cuproptosis marker) compared with si-NC group (p<0.05), with effective CP knockdown verified; the rescue experiment (si-CP+oe-STPG3-AS1) reversed these upregulations, with protein expression restored to within 80% of the control group. MitoSOX Red flow cytometry showed that si-CP treatment increased mtROS positive rate by ≥40% (p<0.05), and oe-STPG3-AS1 overexpression reduced mtROS level to within 75% of the control, confirming the regulatory effect of CP-STPG3-AS1 axis on cuproptosis-related oxidative stress. CCK-8 assay indicated that si-CP treatment induced a ≥30% cell proliferation inhibition rate at 72 h (p<0.05), and the proliferation inhibition rate was reduced to ≤15% in the si-CP+oe-STPG3-AS1 group, demonstrating that STPG3-AS1 mediated the inhibitory effect of CP knockdown on 22Rv1 cell proliferation. Consistent trends were observed in the si-SLC31A1 and si-SLC31A1+oe-LIPE-AS1 groups (**Figure 6**).

**Figure 6:**
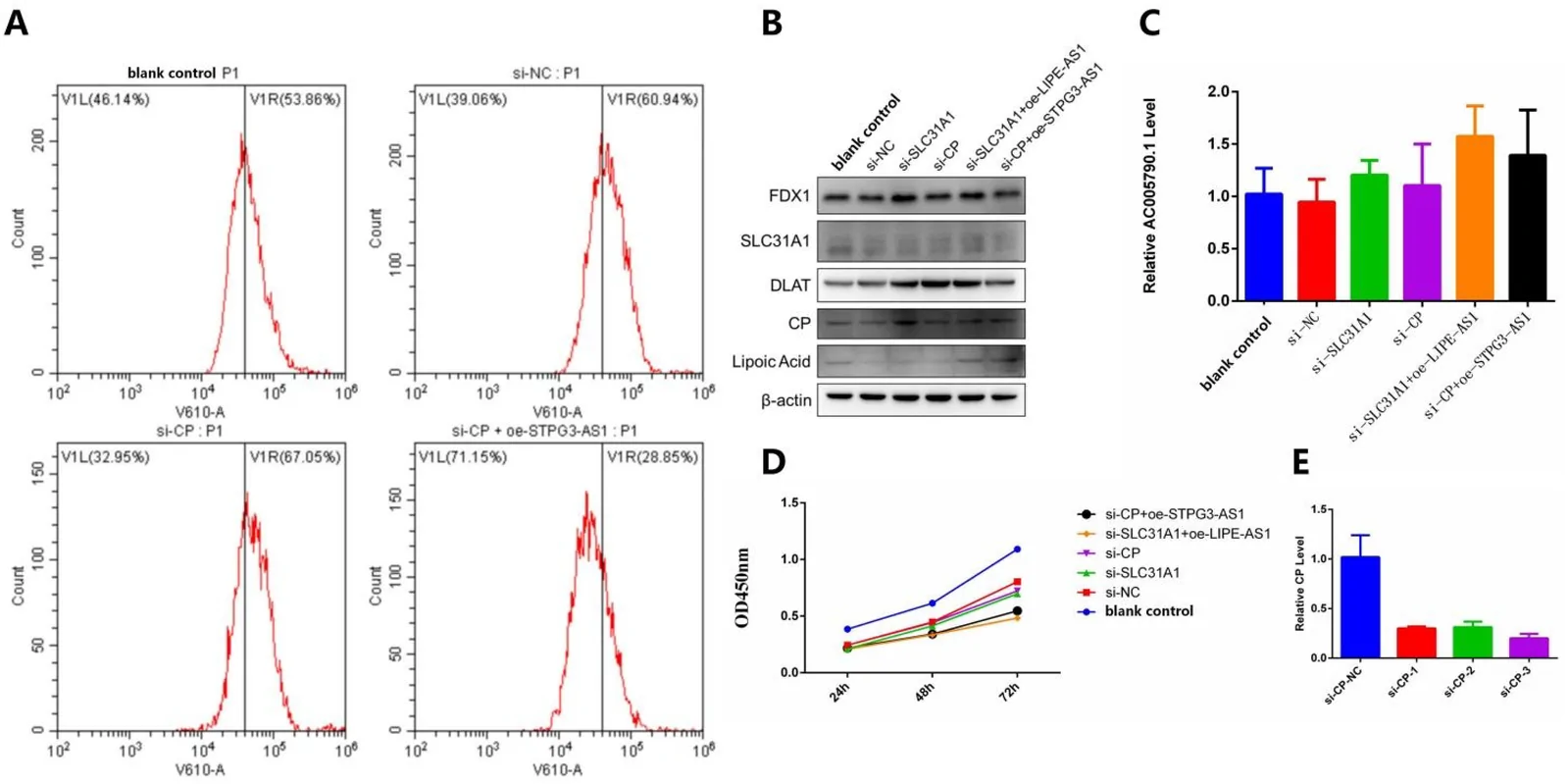
Functional and molecular assessment of CP-mediated regulation of cuproptosis-associated molecular changes in 22Rv1 cells: **(A)** MitoSOX Red flow cytometry: mtROS positive rate in each group, si-CP increased mtROS (≥40%, p<0.05), rescued by oe-STPG3-AS1. **(B)** Western blot (WB) assay: Expression of cuproptosis-related proteins (CP, FDX1, DLAT, lipoylated proteins); si-CP upregulated FDX1/DLAT/lipoylated proteins (≥50%, p<0.05), reversed by oe-STPG3-AS1. **(C)** qRT-PCR assay: Relative expression of LIPE-AS1, AC005790.1 and STPG3-AS1 in each group, si-CP downregulated STPG3-AS1 (≥30%, p<0.05) and si-SLC31A1 downregulated LIPE-AS1/AC005790.1 (≥40%, p<0.05). **(D)** CCK-8 assay: 22Rv1 cell proliferation at 24/48/72 h; si-CP inhibited proliferation (≥30% at 72 h, p<0.05), the effect was attenuated by oe-STPG3-AS1. All experiments were performed in triplicate (n=3), data are mean ± SD, p<0.05 was statistically significant. **(E)** siRNA transfection: CP knockdown efficiency verification by qRT-PCR, si-CP-3 was selected for subsequent experiments.

## 4 Discussion

This study systematically investigated the potential crosstalk between ceruloplasmin (CP) and the cuproptosis pathway in prostate cancer (PCa) through an integrated approach of bioinformatic analysis of the TCGA-PRAD dataset and *in vitro* functional validation in two androgen-independent castration-resistant PCa (CRPC) cell lines (PC-3 and 22Rv1). The core findings revealed 80 intersection genes between CP-associated genes and the cuproptosis-related gene set, with classic cuproptosis core regulators (FDX1, DLST, LIAS, DLD) significantly upregulated in PCa tissues while CP expression showed no differential change (32). We further confirmed a weak but significant positive co-expression pattern between CP and these cuproptosis core genes, and for the first time provided experimental evidence that CP knockdown induces cuproptosis-associated molecular changes in 22Rv1 cells via regulating lncRNA STPG3-AS1, accompanied by altered mitochondrial oxidative stress and inhibited cell proliferation. Exogenous CP treatment also exerted a concentration-dependent inhibitory effect on proliferation-associated metabolic activity and induced apoptosis-related morphological changes in PC-3 cells (33). Collectively, these results fill a critical knowledge gap in the functional association between CP and cuproptosis in PCa, and identify a novel CP-STPG3-AS1 regulatory axis involved in cuproptosis modulation in CRPC cells.

A key observation from the bioinformatic analysis was the distinct expression patterns of CP and classic cuproptosis core genes in PCa tissues: the four core cuproptosis genes were markedly upregulated in PCa, while CP expression showed no statistical difference between tumor and normal tissues. This finding suggests that the dysregulation of the cuproptosis pathway itself, rather than CP transcriptional expression, is closely associated with PCa oncogenesis and progression—consistent with previous reports that cuproptosis core gene aberrations are a hallmark of PCa malignant progression (34). The weak positive correlations between CP and FDX1/DLST/LIAS/DLD (r=0.205-0.300) further indicate a potential co-expression pattern rather than a direct regulatory relationship, which clarifies the earlier misinterpretation of “reprogramming” and aligns with the biological nature of CP as a copper homeostasis regulator that modulates cuproptosis indirectly rather than through direct transcriptional control of core cuproptosis genes. The 80 identified intersection genes were enriched in copper ion homeostasis and cell death regulation, with core PPI network nodes including copper transport-related genes (ATP7A, ATP7B) and oxidative stress regulators (HMOX1, SOD1/2); these genes likely act as key intermediaries in the CP-cuproptosis crosstalk, and their functional characterization will be a focus of future mechanistic research.

The GO/KEGG enrichment and PPI network analyses of the 80 intersection genes further contextualized the biological functions of CP-cuproptosis crosstalk in PCa. The significant enrichment of intersection genes in copper ion homeostasis and copper ion binding directly links these genes to the core biological process of cuproptosis, while enrichment in blood microparticles and secretory granule lumen implies potential involvement in the tumor microenvironment and intercellular communication—processes that are increasingly recognized as critical for cuproptosis modulation in cancer. The top core PPI nodes (ATP7A, ATP7B, HMOX1) are well-known regulators of copper transport and oxidative stress, and their high connectivity in the network suggests that they may form a regulatory module with CP to modulate cuproptosis activity in PCa. For example, ATP7A/B-mediated copper efflux is a key component of cellular copper homeostasis, and its interaction with CP may fine-tune intracellular copper levels to control cuproptosis induction—an interaction that warrants further experimental validation.

The *in vitro* validation in two CRPC cell lines addressed the critical limitations of the original study and provided direct experimental evidence for CP-mediated regulation of cuproptosis-associated molecular changes. In PC-3 cells, exogenous CP treatment reduced proliferation-associated metabolic activity and induced apoptosis-related morphological changes in a concentration-dependent manner, confirming the functional effect of CP on PCa cell behavior; however, the mild inhibitory effect of CP on PC-3 cells also suggests that exogenous CP alone may not be a potent anti-PCa agent, and its biological effects require combination with other pathways or agents for enhancement (35). The more compelling findings came from the 22Rv1 cell knockdown and rescue experiments: si-CP significantly upregulated the expression of cuproptosis-specific markers (FDX1, DLAT, lipoylated proteins) and increased mitochondrial ROS (mtROS) levels, while co-transfection with oe-STPG3-AS1 reversed these changes and restored cell proliferation. This not only supports that CP acts as a negative regulator of cuproptosis-associated molecular changes in CRPC cells but also identifies STPG3-AS1 as a key lncRNA mediator of this regulatory process. The concomitant increase in mtROS upon CP knockdown further links CP regulation to cuproptosis-associated oxidative stress, as mtROS overproduction is a well-characterized downstream event of cuproptosis activation driven by lipoylated protein aggregation and Fe-S protein loss. These results collectively support a functional relationship between CP and cuproptosis-associated molecular changes in PCa, moving beyond the original correlational analysis to provide mechanistic insights into this interaction.

It should be emphasized that the molecular changes observed in 22Rv1 cells—upregulation of FDX1, DLAT and lipoylated proteins together with increased mitochondrial ROS—correspond to the molecular signature of cuproptosis sensitization: FDX1 is the master regulator that promotes lipoylation and reduces Cu(II) to Cu(I), and lipoylated TCA enzymes (e.g., DLAT) are the direct substrates whose copper-mediated oligomerization triggers mitochondrial dysfunction and proteotoxic cell death (36–38). The concurrent elevation of mtROS is consistent with mitochondrial damage downstream of lipoylated-protein aggregation and Fe–S protein loss. We therefore interpret our data as evidence that CP knockdown shifts CRPC cells toward a cuproptosis-sensitized state, rather than as proof of completed cuproptosis execution. Definitive classification of cuproptosis, per the criteria established by Tsvetkov and colleagues, additionally requires demonstration of copper-chelator rescue (e.g., tetrathiomolybdate or D-penicillamine), FDX1 dependence, DLAT oligomerization and Fe-S protein loss; these experiments are ongoing in our laboratory and will be reported separately. Notably, the inclusion of SLC31A1 (CTR1) knockdown—the principal copper-import transporter—provides a complementary copper-limiting perturbation: the parallel phenotypic trends observed under si-SLC31A1 support the copper-dependency of the CP-STPG3-AS1 effects, although they do not substitute for direct chelation experiments.

Several non-mutually-exclusive mechanisms may explain how STPG3-AS1 couples CP-regulated copper homeostasis to lipoylation and cuproptosis. First, as a cytoplasmic lncRNA, STPG3-AS1 may act as a competing endogenous RNA, sponging microRNAs that target FDX1, LIAS, LIPT1, DLD or DLAT, thereby stabilizing cuproptosis-core transcripts; cuproptosis-related lncRNAs in PCa have been shown to function through such ceRNA networks (39). Second, STPG3-AS1 may modulate the expression of copper transporters or lipoylation enzymes in cis or in trans via chromatin-remodeling or promoter-interacting mechanisms, as described for other antisense lncRNAs in PCa (40). Third, given that CP knockdown downregulates STPG3-AS1 and that STPG3-AS1 overexpression reverses all CP-knockdown phenotypes (FDX1, DLAT, lipoylated proteins, mtROS and proliferation), STPG3-AS1 may operate downstream of CP as an effector that transmits copper-homeostatic signals to the mitochondrial lipoylation machinery; the rescue results suggest that STPG3-AS1 is both necessary and, at the expression level, sufficient to mediate these CP-regulated cuproptosis-associated changes. Definitive mapping of the STPG3-AS1 interactome (RNA pulldown and RIP-seq) and its miRNA partners will be required to distinguish these models, and constitutes a central objective of our ongoing work.

Cuproptosis is mechanistically and morphologically distinct from other regulated cell death modalities. Unlike apoptosis—characterized by caspase activation, nuclear condensation/fragmentation, membrane blebbing and apoptotic-body formation—cuproptosis proceeds independently of caspases and does not exhibit classical nuclear fragmentation; its hallmarks are copper-dependent oligomerization of lipoylated TCA enzymes, Fe-S cluster protein loss, mitochondrial shrinkage and proteotoxic stress (41). Ferroptosis is driven by iron-dependent lipid peroxidation and is counteracted by GPX4 (42), necroptosis requires RIPK1/RIPK3/MLKL signaling, and pyroptosis is mediated by gasdermin cleavage (43). In the present study, the two cell models captured distinct, complementary aspects of this spectrum: in PC-3 cells, exogenous CP induced apoptosis-related morphology (nuclear condensation/fragmentation on Hoechst staining) together with reduced metabolic activity, whereas in 22Rv1 cells CP knockdown elicited cuproptosis-associated molecular changes (FDX1/DLAT/lipoylated protein upregulation and mtROS elevation). These observations suggest that CP may engage both modalities depending on cellular context, and that cuproptosis and apoptosis are not mutually exclusive—copper overload can secondarily engage mitochondrial apoptotic pathways (44). Accordingly, we use the term “cuproptosis-associated molecular changes” for the 22Rv1 findings to maintain evidence-based terminology, and definitive cuproptosis identification (chelator rescue and FDX1 dependence) remains a priority for subsequent validation.

Notably, Kaplan-Meier and Cox regression analyses in this study found no statistically significant association between CP/cuproptosis core genes and PCa overall survival, indicating that none of these genes serve as independent prognostic biomarkers for PCa patients. This finding corrects the overstatement of clinical prognostic significance in the original study and highlights two critical considerations for future prognostic research: first, the absence of clinical covariate adjustment (e.g., age, tumor stage, Gleason score) in the survival analysis may reduce the robustness of the results, and second, single-gene prognostic value is limited in PCa, a heterogeneous malignancy, and multi-gene signatures incorporating CP and cuproptosis core genes may have more predictive power (45, 46). While serum copper levels have been reported as a potential prognostic indicator in solid tumors, the lack of differential CP expression in PCa tissues suggests that CP may not be a tissue-specific prognostic marker, and its serum level and functional activity may be more relevant to PCa clinical outcomes—an area requiring further investigation in large clinical cohorts.

This study has several acknowledged limitations that should be considered when interpreting the results, and these limitations also point to directions for future research (47–49). First, *in vitro* experiments were limited to two androgen-independent CRPC cell lines (PC-3 and 22Rv1), and no androgen-dependent PCa cell lines (e.g., LNCaP) or primary PCa cells were used; the results thus cannot be generalized to all PCa subtypes, and future studies should validate the CP-STPG3-AS1 axis in a broader panel of PCa cell models. Second, intracellular copper concentrations were not directly measured in the present study. It should be noted that PC-3 and 22Rv1 cells were cultured under defined conditions in which exogenous copper is delivered predominantly through serum proteins (including CP) in fetal bovine serum, and that the SLC31A1 (CTR1) knockdown arm partially addresses copper-import dependence; nevertheless, direct quantification of intracellular labile copper—rather than total copper, which is largely sequestered by metallothioneins and glutathione and may not reflect the labile Cu(I) pool relevant to cuproptosis (50)—is required to formally link CP regulation to cellular copper availability. Future studies using ICP-MS in combination with labile-copper probes (e.g., CS1) after CP knockdown or overexpression, together with copper-chelator rescue experiments, will be essential to complete the mechanistic picture. Third, survival analysis was based solely on the TCGA-PRAD dataset without clinical covariate adjustment or external validation in an independent cohort; future prognostic research should integrate clinical parameters and multi-center data to evaluate the potential value of the CP-STPG3-AS1 axis as a prognostic signature. Fourth, no *in vivo* animal experiments were performed to validate the functional effects of the CP-STPG3-AS1 axis, and the anti-tumor efficacy of targeting this axis in combination with cuproptosis inducers remains unknown. Finally, the upstream signaling pathways that regulate CP expression and the downstream targets of STPG3-AS1 in the cuproptosis pathway were not explored; identifying these regulatory elements will be essential to fully characterize the CP-STPG3-AS1-cuproptosis regulatory network in PCa (51–53).

Despite these limitations, this study provides important preliminary insights into the crosstalk between CP and cuproptosis in PCa and identifies a novel lncRNA-mediated regulatory axis with potential therapeutic implications for CRPC (54). Future research should focus on three key directions: first, validating the CP-STPG3-AS1 axis in *in vivo* PCa xenograft models and exploring its therapeutic potential in combination with cuproptosis inducers (e.g., elesclomol) or standard-of-care chemotherapies (e.g., docetaxel) for CRPC; second, investigating the expression and clinical correlation of CP and STPG3-AS1 in large PCa clinical cohorts, including serum and tissue samples, to evaluate their potential as diagnostic or predictive biomarkers; third, elucidating the molecular mechanism by which STPG3-AS1 modulates cuproptosis core genes and mtROS levels, and identifying its direct RNA or protein targets. Additionally, exploring the interaction between CP and other copper homeostasis regulators (e.g., ATP7A/B, CTR1) in cuproptosis modulation will provide a more comprehensive understanding of the role of systemic copper metabolism in PCa progression.

## 5 Conclusions

This study supports that STPG3-AS1 mediates ceruloplasmin-regulated lipoylation and cuproptosis-associated molecular changes in castration-resistant prostate cancer, and clarifies the core regulatory axis formed by STPG3-AS1 and ceruloplasmin underlying these cuproptosis-associated molecular changes. The findings reveal a novel functional link between CP and cuproptosis-associated molecular changes in PCa, and identify the STPG3-AS1-CP axis as a potential new target for CRPC therapy. While further mechanistic and *in vivo* validation is required, this study lays a critical foundation for future research into the role of copper homeostasis regulators in cuproptosis modulation, and provides a new perspective for developing cuproptosis-based targeted therapies for CRPC.

## Data Availability

The cuproptosis-related gene set was obtained from GSEA/MSigDB (https://www.gsea-msigdb.org/ gsea/msigdb; GPL11670, GPL14663) and published collections, with annotations from GeneCards (https://www.genecards.org/). CP-associated genes were retrieved from GeneCards and STRING (https://string-db.org/). The TCGA-PRAD dataset was accessed via UCSC Xena (https://xenabrowser.net/datapages/?dataset=TCGA-PRAD&host=https://tcga.xenahubs.net; dbGaP: phs000178). In vitro experimental data are available from the corresponding author upon reasonable request.

## References

[1] Wu J He J Liu Z Zhu X Li Z Chen A . Cuproptosis: mechanism, role, and advances in urological malignancies. Med Res Rev. (2024) 44:1662–82. doi: 10.1002/med.22025 38299968

[2] Cheng B Tang C Xie J Zhou Q Luo T Wang Q . Cuproptosis illustrates tumor micro-environment features and predicts prostate cancer therapeutic sensitivity and prognosis. Life Sci. (2023) 325:121659. doi: 10.1016/j.lfs.2023.121659 37011878

[3] Bray F Laversanne M Sung H Ferlay J Siegel RL Soerjomataram I . Global cancer statistics 2022: GLOBOCAN estimates of incidence and mortality worldwide for 36 cancers in 185 countries. CA Cancer J Clin. (2024) 74:229–63. doi: 10.3322/caac.21834 38572751

[4] Siegel RL Kratzer TB Wagle NS Sung H Jemal A . Cancer statistics, 2026. CA Cancer J Clin. (2026) 76:e70043. doi: 10.3322/caac.70043 41528114 PMC12798275

[5] Tannock IF de Wit R Berry WR Horti J Pluzanska A Chi KN . Docetaxel plus prednisone or mitoxantrone plus prednisone for advanced prostate cancer. N Engl J Med. (2004) 351:1502–12. doi: 10.1056/nejmoa040720 15470213

[6] Spratt DE Srinivas S Adra N Ahmed B An Y Bitting R . Prostate cancer, version 3.2026, NCCN clinical practice guidelines in oncology. J Natl Compr Canc Netw. (2025) 23:469–93. doi: 10.6004/jnccn.2025.0052 41213253

[7] Scher HI Fizazi K Saad F Taplin ME Sternberg CN Miller K . Increased survival with enzalutamide in prostate cancer after chemotherapy. N Engl J Med. (2012) 367:1187–97. doi: 10.1056/nejmoa1207506 22894553

[8] Belge Bilgin G Bilgin C Packard AT Burkett BJ Oztekin ES Thorpe MP . PSMA-RECAP: [177Lu]Lu-PSMA-617 retreatment in metastatic castration-resistant prostate cancer. Front Oncol. (2026) 16:1831393. doi: 10.3389/fonc.2026.1831393 42729213 PMC13561789

[9] Hofman MS Emmett L Sandhu S Iravani A Joshua AM Goh JC . 177Lu]Lu-PSMA-617 versus cabazitaxel in patients with metastatic castration-resistant prostate cancer (TheraP): a randomised, open-label, phase 2 trial. Lancet. (2021) 397:797–804. doi: 10.1016/s0140-6736(21)00237-3 33581798

[10] He JW Li PZ Huang ZX . Copper death combination therapy: the innovative frontier and challenges in prostate cancer treatment. Cancer Biol Ther. (2025) 26:2532224. doi: 10.1080/15384047.2025.2532224 40660923 PMC12269702

[11] He JW Li PZ . Lipid biomarkers of prostate cancer: insights from triglycerides and saturated fatty acid metabolism. Future Oncol. (2026) 22:2279–86. doi: 10.1080/14796694.2026.2707002 42489496 PMC13540192

[12] Wen H Qu C Wang Z Gao H Liu W Wang H . Cuproptosis enhances docetaxel chemosensitivity by inhibiting autophagy via the DLAT/mTOR pathway in prostate cancer. FASEB Journal: Off Publ Fed Am Societies For Exp Biol. (2023) 37:e23145. doi: 10.1096/fj.202300980r 37584654

[13] Cheng X Zeng Z Yang H Chen Y Liu Y Zhou X . Novel cuproptosis-related long non-coding RNA signature to predict prognosis in prostate carcinoma. BMC Cancer. (2023) 23:105. doi: 10.1186/s12885-023-10584-0 36717792 PMC9887873

[14] Zhong H Lai Y Ouyang W Yu Y Wu Y He X . Integrative analysis of cuproptosis-related lncRNAs: unveiling prognostic significance, immune microenvironment, and copper-induced mechanisms in prostate cancer. Cancer Pathogenesis Ther. (2024) 3:48–59. doi: 10.1016/j.cpt.2024.03.004 PMC11764251 39872368

[15] Bontempo NJS Paixão DA Lima PMAP Barros DCT Borges DS Orsolin PC . Copper(II) complex containing 4-fluorophenoxyacetic acid hydrazide and 1,10-phenanthroline: a prostate cancer cell-selective and low-toxic copper(II) compound. Molecules (Basel Switzerland). (2022) 27:7097. doi: 10.3390/molecules27207097 36296690 PMC9609220

[16] Alhasawi MAI Aatif M Muteeb G Alam MW Oirdi ME Farhan M . Curcumin and its derivatives induce apoptosis in human cancer cells by mobilizing and redox cycling genomic copper ions. Molecules (Basel Switzerland). (2022) 27:7410. doi: 10.3390/molecules27217410 36364236 PMC9659251

[17] Jin L Mei W Liu X Sun X Xin S Zhou Z . Identification of cuproptosis-related subtypes, the development of a prognosis model, and characterization of tumor microenvironment infiltration in prostate cancer. Front Immunol. (2022) 13:974034. doi: 10.3389/fimmu.2022.974034 36203594 PMC9530990

[18] Ding Y Feng Y Huang Z Zhang Y Li X Liu R . SOX15 transcriptionally increases the function of AOC1 to modulate ferroptosis and progression in prostate cancer. Cell Death Dis. (2022) 13:673. doi: 10.1038/s41419-022-05108-w 35922412 PMC9349193

[19] Yu Z Deng H Chao H Song Z Zeng T . Construction of a cuproptosis-related lncRNA signature to predict biochemical recurrence of prostate cancer. Oncol Lett. (2024) 28:526. doi: 10.3892/ol.2024.14659 39268161 PMC11391508

[20] Li X Wang S Wei Y Xie C Chen Y Wu F . AR-targeted therapies sensitize prostate cancer to cuproptosis by transcriptionally activating FDX1. Proc Natl Acad Sci USA. (2026) 123:e2534978123. doi: 10.1073/pnas.2534978123 42066048 PMC13142982

[21] Tralongo P Ballato M Fiorentino V Giordano WG Zuccalà V Pizzimenti C . Cuproptosis: a review on mechanisms, role in solid and hematological tumors, and association with viral infections. Mediterr J Hematol Infect Dis. (2025) 17:e2025052. doi: 10.4084/MJHID.2025.052 PMC12240258 40636277

[22] Machado JF Sequeira D Marques F Piedade MFM Villa de Brito MJ Helena Garcia M . New copper(I) complexes selective for prostate cancer cells. Dalton Trans (Cambridge Engl 2003). (2020) 49:12273–86. doi: 10.1039/d0dt02157a 32839796

[23] Wang X Xu C Ma J Wang X Chen X . Identification of key genes participating in copper-diethyldithiocarbamate-related cell death process and predicting the development of prostate cancer. Discover Oncol. (2024) 15:519. doi: 10.1007/s12672-024-01390-6 39361158 PMC11450124

[24] He JW Li PZ Huang ZX . Bispecific antibodies in metastatic castration-resistant prostate cancer: therapeutic strategies and frontier advances. World J Urol. (2025) 43:694. doi: 10.1007/s00345-025-06075-5 41236620

[25] Zhou G Chen C Wu H Lin J Liu H Tao Y . LncRNA AP000842.3 triggers the malignant progression of prostate cancer by regulating cuproptosis related gene NFAT5. Technol Cancer Res Treat. (2024) 23:15330338241255585. doi: 10.1177/15330338241255585 38780486 PMC11119409

[26] Gao X Zhao H Liu J Wang M Dai Z Hao W . Enzalutamide sensitizes castration-resistant prostate cancer to copper-mediated cell death. Advanced Sci (Weinheim Baden-Wurttemberg Germany). (2024) 11:e2401396. doi: 10.1002/advs.202401396 38859590 PMC11321675

[27] Farhan M Rizvi A Ali F Ahmad A Aatif M Malik A . Pomegranate juice anthocyanidins induce cell death in human cancer cells by mobilizing intracellular copper ions and producing reactive oxygen species. Front Oncol. (2022) 12:998346. doi: 10.3389/fonc.2022.998346 36147917 PMC9487716

[28] Tsvetkov P Coy S Petrova B Dreishpoon M Verma A Abdusamad M . Copper induces cell death by targeting lipoylated TCA cycle proteins. Science. (2022) 375:1254–61. doi: 10.1126/science.abf0529 35298263 PMC9273333

[29] Chen X Li H Xu C Wang X Wu G Li C . CYP19A1 is downregulated by BRD4 and suppresses castration-resistant prostate cancer cell invasion and proliferation by decreasing AR expression. Am J Cancer Res. (2023) 13:4003–20. PMC10560923 37818065

[30] Chen X Ma J Xu C Wang L Yao Y Wang X . Identification of hub genes predicting the development of prostate cancer from benign prostate hyperplasia and analyzing their clinical value in prostate cancer by bioinformatic analysis. Discover Oncol. (2022) 13:54. doi: 10.1007/s12672-022-00508-y 35768705 PMC9243208

[31] Chen X Li H Liu B Wang X Zhou W Wu G . Identification and validation of MSMB as a critical gene for prostate cancer development in obese people. Am J Cancer Res. (2023) 13:1582–93. PMC10164797 37168344

[32] Ge EJ Bush AI Casini A Cobine PA Cross JR DeNicola GM . Connecting copper and cancer: from transition metal signalling to metalloplasia. Nat Rev Cancer. (2022) 22:102–13. doi: 10.1038/s41568-021-00417-2 PMC8810673 34764459

[33] Wang XY Xiao J Ma HQ . Two new Cu(II)-based coordination polymers: inhibitory activity on prostate cancer by reducing EGF-R expression and HIPPO signaling pathway activation. J Biomater Sci Polym Ed. (2020) 31:1741–55. doi: 10.1080/09205063.2020.1775057 32529956

[34] Zehra S Gomez-Ruiz S Siddique HR Tabassum S Arjmand F . Water soluble ionic co(II), Cu(II) and zn(II) diimine-glycinate complexes targeted to tRNA: structural description, *in vitro* comparative binding, cleavage and cytotoxic studies towards chemoresistant prostate cancer cells. Dalton Trans. (2020) 49:16830–48. doi: 10.1039/d0dt02657c 33179662

[35] Merrick MJ Rotsch DA Tiwari A Nolen J Brossard T Song J . Imaging and dosimetric characteristics of (67) Cu. Phys Med Biol. (2021) 66:035002. doi: 10.1088/1361-6560/abca52 33496267

[36] Tang D Chen X Kroemer G . Cuproptosis: a copper-triggered modality of mitochondrial cell death. Cell Res. (2022) 32:417–8. doi: 10.1038/s41422-022-00653-7 35354936 PMC9061796

[37] Bordini J Morisi F Elia AR Santambrogio P Pagani A Cucchiara V . Iron induces cell death and strengthens the efficacy of antiandrogen therapy in prostate cancer models. Clin Cancer Res. (2020) 26:6387–98. doi: 10.1158/1078-0432.ccr-20-3182 32928793

[38] Campbell KJ Leung HY . Evasion of cell death: a contributory factor in prostate cancer development and treatment resistance. Cancer Lett. (2021) 520:213–21. doi: 10.1016/j.canlet.2021.07.045 34343635

[39] He JW Huang ZX Su YF Mo YQ . Dietary saturated fatty acids and prostate cancer: insights into NF-κB pathway and lipid metabolism mechanisms. Discov Oncol. (2025) 16:1166. doi: 10.1007/s12672-025-03005-0 40542303 PMC12181547

[40] Zubair H Azim S Khan HY Ullah MF Wu D Singh AP . Mobilization of intracellular copper by gossypol and apogossypolone leads to reactive oxygen species-mediated cell death: putative anticancer mechanism. Int J Mol Sci. (2016) 17:973. doi: 10.3390/ijms17060973 27331811 PMC4926505

[41] Wang Y Zeng S Lin TM Krugner-Higby L Lyman D Steffen D . Evaluating the anticancer properties of liposomal copper in a nude xenograft mouse model of human prostate cancer: formulation, *in vitro*, *in vivo*, histology and tissue distribution studies. Pharm Res. (2014) 31:3106–19. doi: 10.1007/s11095-014-1403-6 24848339 PMC4214917

[42] Demirci S Doğan A Türkmen NB Telci D Çağlayan AB Beker MÇ . Poloxamer P85 increases anticancer activity of schiff base against prostate cancer *in vitro* and *in vivo*. Anti-Cancer Drugs. (2017) 28:869–79. doi: 10.1097/cad.0000000000000528 28614092

[43] Chen D Peng F Cui QC Daniel KG Orlu S Liu J . Inhibition of prostate cancer cellular proteasome activity by a pyrrolidine dithiocarbamate-copper complex is associated with suppression of proliferation and induction of apoptosis. Front Bioscience: A J Virtual Library. (2005) 10:2932–9. doi: 10.2741/1749 15970547

[44] Milacic V Jiao P Zhang B Yan B Dou QP . Novel 8-hydroxylquinoline analogs induce copper-dependent proteasome inhibition and cell death in human breast cancer cells. Int J Oncol. (2009) 35:1481–91. doi: 10.3892/ijo_00000467 PMC3779835 19885572

[45] Chen D Cui QC Yang H Dou QP . Disulfiram, a clinically used anti-alcoholism drug and copper-binding agent, induces apoptotic cell death in breast cancer cultures and xenografts via inhibition of the proteasome activity. Cancer Res. (2006) 66:10425–33. doi: 10.1158/0008-5472.can-06-2126 17079463

[46] Chen W Yang W Chen P Huang Y Li F . Disulfiram copper nanoparticles prepared with a stabilized metal ion ligand complex method for treating drug-resistant prostate cancers. ACS Appl Materials Interfaces. (2018) 10:41118–28. doi: 10.1021/acsami.8b14940 30444340

[47] Doğan A Demirci S Türkmen NB Çağlayan AB Aydın S Telci D . Schiff base - poloxamer P85 combination prevents prostate cancer progression in C57/Bl6 mice. Prostate. (2016) 76:1454–63. doi: 10.1002/pros.23229 27338565

[48] Demirci S Doğan A Türkmen NB Telci D Rizvanov AA Şahin F . Schiff base - poloxamer P85 combination demonstrates chemotherapeutic effect on prostate cancer cells *in vitro*. Biomedicine Pharmacotherapy = Biomedecine Pharmacotherapie. (2017) 86:492–501. doi: 10.1016/j.biopha.2016.11.101 28012929

[49] Duan L Shen H Zhao G Yang R Cai X Zhang L . Inhibitory effect of disulfiram/copper complex on non-small cell lung cancer cells. Biochem Biophys Res Commun. (2014) 446:1010–6. doi: 10.1016/j.bbrc.2014.03.047 24657266

[50] Tsvetkov P Coy S Petrova B Dreishpoon M Verma A Abdusamad M . Erratum for the research article "Copper induces cell death by targeting lipoylated TCA cycle proteins," by P. Tsvetkov et al. Sci (New York NY). (2022) 376:eabq4855. doi: 10.1126/science.abq4855 35446667

[51] Chen Y Liao L Wang B Wu Z . Identification and validation of immune and cuproptosis - related genes for diabetic nephropathy by WGCNA and machine learning. Front Immunol. (2024) 15:1332279. doi: 10.3389/fimmu.2024.1332279 38390317 PMC10881670

[52] He JW Li PZ Huang ZX . TMEM16A in prostate cancer: mechanistic insights and therapeutic implications. Cancer Cell Int. (2025) 25:278. doi: 10.1186/s12935-025-03914-8 40707942 PMC12288284

[53] Sramkoski RM Pretlow TG 2nd Giaconia JM Pretlow TP Schwartz S Sy MS . A new human prostate carcinoma cell line, 22Rv1. In Vitro Cell Dev Biol Anim. (1999) 35:403–9. doi: 10.1007/s11626-999-0115-4 10462204

[54] van Bokhoven A Varella-Garcia M Korch C Johannes WU Smith EE Miller HL . Molecular characterization of human prostate carcinoma cell lines. Prostate. (2003) 57:205–25. doi: 10.1002/pros.10290 14518029

